# Genome Sequence of *E. coli* O104:H4 Leads to Rapid Development of a Targeted Antimicrobial Agent against This Emerging Pathogen

**DOI:** 10.1371/journal.pone.0033637

**Published:** 2012-03-14

**Authors:** Dean Scholl, Dana Gebhart, Steven R. Williams, Anna Bates, Robert Mandrell

**Affiliations:** 1 AvidBiotics Corporation, South San Francisco, California, United States of America; 2 Produce Safety and Microbiology Unit, Agricultural Research Service, Western Regional Research Center, United States Department of Agriculture, Albany, California, United States of America; University of Massachusetts Medical School, United States of America

## Abstract

A recent widespread outbreak of *Escherichia coli* O104:H4 in Germany demonstrates the dynamic nature of emerging and re-emerging food-borne pathogens, particularly STECs and related pathogenic *E. coli*. Rapid genome sequencing and public availability of these data from the German outbreak strain allowed us to identify an O-antigen-specific bacteriophage tail spike protein encoded in the genome. We synthesized this gene and fused it to the tail fiber gene of an R-type pyocin, a phage tail-like bacteriocin, and expressed the novel bacteriocin such that the tail fiber fusion was incorporated into the bacteriocin structure. The resulting particles have bactericidal activity specifically against *E. coli* strains that produce the O104 lipopolysaccharide antigen, including the outbreak strain. This O-antigen tailspike-R-type pyocin strategy provides a platform to respond rapidly to emerging pathogens upon the availability of the pathogen's genome sequence.

## Introduction

Shiga toxin producing *E. coli* (STECs) cause foodborne outbreaks of hemorrhagic colitis which often leads to hemolytic-uremic syndrome (HUS) [1]. The highly virulent *E. coli* O157:H7 has been the most concerning strain in recent years and has been declared an adulterant in food products [2], [3]. However, it has long been recognized that non-O157 STECs are also prevalent, and six additional STECs have also recently been declared adulterants [4]. More recently a very large outbreak in Germany was caused by a highly virulent enteroaggregative *E. coli* O104:H4 that encodes the potent shiga toxin 2, resulting in a high incidence of HUS [5]–[10].

R-type pyocins are high molecular weight bacteriocins produced by some *Pseudomonas aeruginosa* strains and have bactericidal activity mainly against other strains within the species, for a review see [11]. These bacteriocins resemble the contractile tail structures of phages of the *Myoviridae* family, particularly the P2-like phages. Upon binding to a receptor on the target bacterium surface via six homotrimeric tail fibers, the R-type pyocin structure contracts its sheath, extending its core to form a channel across the bacterial envelope. As a consequence, the bacterial membrane potential is dissipated, resulting in rapid death of the cell [12]. The binding of a single pyocin particle can kill a bacterium [13]. R-type pyocins can be retargeted to other bacteria by creating fusions between the pyocin tail fiber and tail fibers or tail spikes (i.e. receptor binding proteins) from bacteriophages specific for the target bacteria, thereby changing the specific interaction near the surface of the outer membrane [14], [15]. One such engineered pyocin, AvR2-V10, has been retargeted to specifically kill *E. coli* O157:H7 by fusing the O-antigen specific tail spike from phage phiV10 to the R2 pyocin tail fiber. AvR2-V10.3 has been shown to be efficacious in preventing and treating *E. coli* O157:H7-induced hemorrhagic colitis in a rabbit model and represents a new class of targeted antimicrobials [16].

Here we created an O104-specific R-type pyocin that kills the highly virulent shiga toxin-producing enteroaggregative *E. coli* O104:H4 associated with the 2011 outbreak as well as other *E. coli* O104 strains. This was accomplished without any O104 strain in hand or a donor bacteriophage. From the on-line draft genome sequence of *E. coli* O104:H4, we identified a gene encoding an O-antigen specific P22-like tailspike. This gene was then synthesized and used to engineer an appropriate pyocin tail fiber fusion gene that was then expressed and incorporated into the pyocin structure creating AvR2-104.1. AvR2-104.1 is highly sensitive and specific, killing all tested *E. coli* strains that produce the O104 antigen but no other O-antigen type. Thus, the rapid acquisition and public availability of the genome sequence allowed us to identify a strategy to develop a targeted antibacterial to this emerging pathogen.

## Results

**Figure 1:**
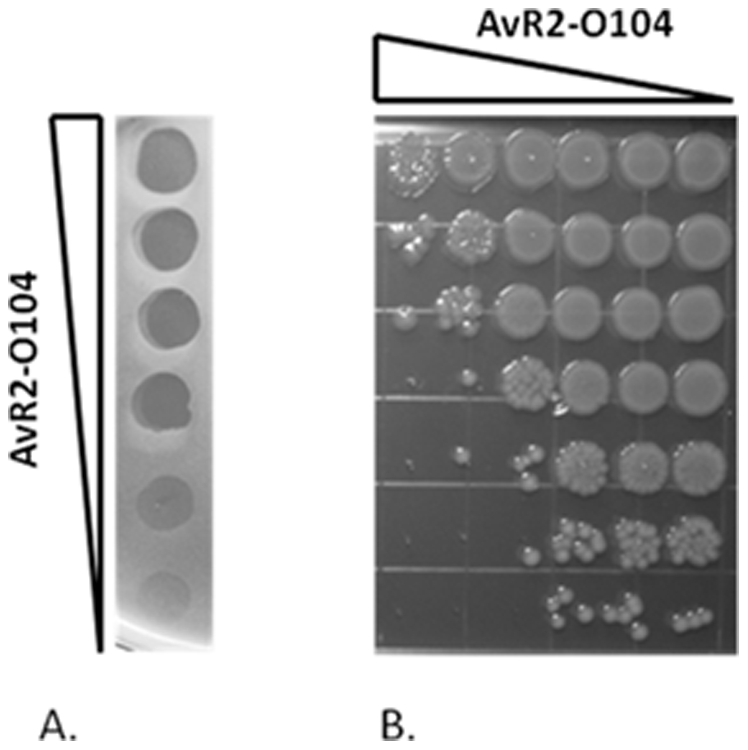
Bactericidal activity of AvR2-O104.1. A. Lawn spot assay of a 5-fold dilution series of AvR2-104 showing zones of killing *E. coli* strain RM13368. B. Titration survival assay of AvR2-104 on strain RM13368. 10^8^ cells were incubated in individual wells of a multi-well plate with 5-fold serial dilutions of pyocin (left to right) for a period of 40 minutes. Survivors are then 10-fold serially diluted (top to bottom) and plated on LB agar to determine the number of surviving colony forming bacteria. By this assay a typical 200 ml preparation of AvR2-104 yields approximately 10^12^ killing units total (see Materials and Methods).

**Figure 2:**
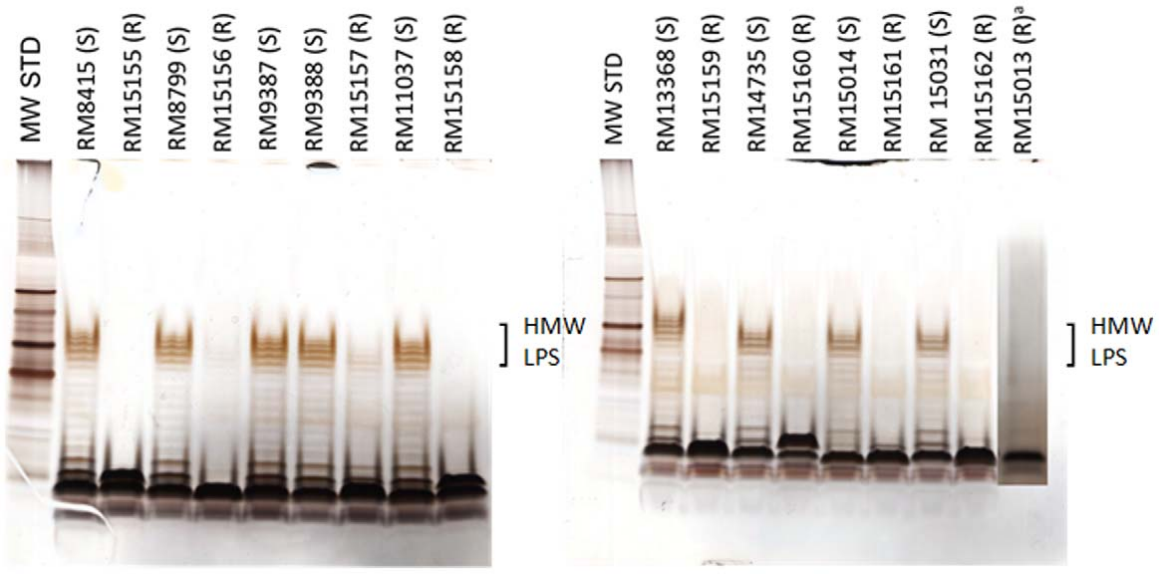
Silver stained SDS-PAGE analysis of LPS extracted from *E. coli* O104 strains and resistant mutants. See Tables 1 and 2 for strain details. The pyocin-resistant strains (R) express a highly truncated O-antigen, or O-antigen that have a decreased concentration of the high molecular weight structure (HMW LPS) compared to the pyocin-sensitive strains (S). ^a^Strain RM15013 was run in a different gel than the other strains shown in this panel.

BLAST search analysis of the draft genome sequence of *E. coli* O104:H4 from the German outbreak strain revealed a P22-like bacteriophage tailspike protein, locus EGR74047, from strain LB226692. This entire gene was synthesized and then used as a template to make fusions with the R2 pyocin tail fiber. Two fusions were made between the first 164 amino acids of the R2 pyocin tail fiber and amino acids 114–659 or amino acids 118–659 of the *E. coli* O104 tail spike. The genes for these two fusions were separately cloned into plasmid DG100 under transcriptional control of P_TAC_ and separately transformed into *P. aeruginosa* strain PAO1 *Δprf15*, deleted for the R2 pyocin wild type tail fiber gene [14], [15]. This system is designed such that the tail fiber fusions supplied *in trans* complement the tail fiber deletion of the genome-encoded pyocin gene cluster. Upon induction with IPTG (to induce the fusions) and mitomycin C (to induce the pyocin gene cluster), R-type pyocin particles were produced that incorporated the tail fiber fusions, termed AvR2-O104.1 and AvR2-O104.2. Both particles were initially tested against *E. coli* strain RM13368 and were found to have the predicted bactericidal activity. AvR2-O104.1 was chosen for further characterization. Figure 1A shows the results of a lawn spot assay of different dilutions of AvR2-O104.1 against strain RM13368. Clear zones of killing occurred at the highest concentrations of the pyocin, with zones of clearing becoming somewhat more opaque with decreasing concentrations of pyocin. The results of a titration survival assay show multiple log reductions in bacterial counts with bactericidal activity decreasing with decreasing pyocin concentration (Figure 2B). Based on the survival assay, yield from a 200 ml culture was about was about 10^12^ killing units (KU).

The bactericidal activity of AvR2-104.1 was evaluated with multiple *E. coli* O104 isolates: five O104 strains obtained from cattle, an O104:H21 clinical outbreak strain, O104:H4 enteroaggregative strain (EAEC) 55989, and five strains from four patients associated with the 2011 German outbreak. All strains that expressed the O104 antigen were sensitive to AvR2-O104.1 (Table 1). A group of seven non-O104 serotypes was also tested to evaluate the specificity of the pyocin (Table 2). One *E. coli* strain, serotype O9:K9:H12 (ATCC 23505) was also sensitive to AvR-O104.1, a result consistent with the fact that K9 high molecular weight polysaccharide capsule is structurally identical to the repeating sugar subunit of the O104 high molecular weight lipopolysaccharide (LPS) [17]. However, none of the six other non-O104 strains of *E. coli* was sensitive to AvR2-O104.1. It is noteworthy that an isolate from a culture plate of a stool sample US patient (“patient 3”) associated with the German outbreak was insensitive (Table 2, strain RM15013). However, we determined subsequently that this isolate is a phenotypic variant compared to the other German outbreak strains, and similar to “rough” LPS mutants that lack high molecular weight repeating units of O-antigen (Table 2, Figure 2).

**Table 1:** *E. coli* strains sensitive to AvR2-O104.1.

Strain Number	Serotype/Serovar	Details^a^
**RM8415**	O104	Cow feces, CA, 2009
**RM8799**	O104	Cow feces, CA, 2009
**RM9387**	O104	Cow feces, CA, 2009
**RM9388**	O104	Cow feces, CA, 2009
**RM11037**	O104	Cow feces, CA, 2010
**RM13368**	O104:H21	CDC 94-3024; ATCC BAA-178
**RM14735**	O104:H4	German outbreak, US-MA patient 1
**RM15012**	O104:H4	German outbreak,US-MI patient 2
**RM15014**	O104:H4	German outbreak, US-MI patient 3
**RM15031**	O104:H4	German outbreak, Canada
**RM15038**	O104:H4	Republic of Georgia, 2009EL-2050, CDC
**RM15039**	O104:H4	Republic of Georgia, 2009EL-2071, CDC
**RM15037**	O104:H4	Enteroaggregative strain 55989
**ATCC 23505**	O9:K9(B):H12	NCDC Bi 316-42, human, peritonitis

a Source, year sample collected, other strain designations, and two letter state abbreviations (CA, California; MA, Massachusetts; MI, Michigan).

**Table 2:** *E. coli* strains resistant to AvR2-O104.1.

Strain number	Serotype/Serovar	Details
**RM8297**	O26	Cow feces
**RM10351**	O111	Cow feces
**RM2048**	O45:H2	DECA 11C, human isolate, hemorrhagic colitis, CDC
**RM5704**	O145:H28	CDPH:Z11198, human isolate
**RM10068**	O121	Water trough (ranch)
**RM6158**	O157:H7	Feral swine feces
**ATCC 43895**	O157:H7	CDC EDL933, hamburger (outbreak)
**RM10744**	O103	Cow feces
**RM15013**	O104:H4	German outbreak, US-MI patient 3, variant PFGE profile (1 band difference), truncated LPS
**RM15155**	O104	Resistant mutant of RM8145
**RM15156**	O104	Resistant mutant of RM8799
**RM15157**	O104	Resistant mutant of RM9388
**RM15158**	O104	Resistant mutant of RM11037
**RM15159**	O104:H21	Resistant mutant of RM13368
**RM15160**	O104:H4	Resistant mutant of RM14735
**RM15161**	O104:H4	Resistant mutant of RM15014
**RM15162**	O104:H4	Resistant mutant of RM15031

Surviving colonies sometimes arise within the zone of clearing in a bacterial lawn of an R-type pyocin spot assay, and often prove to be resistant mutants. One or more such isolated colonies were picked from several of the O104 strains, subcultured, and subjected to further testing (see Table 2). LPS isolated from those confirmed to be resistant to AvR2-O104.1 were analyzed by SDS-PAGE (Figure 2). Visual analysis of the gels revealed that bands corresponding to the high molecular weight LPS of the mutants were missing or greatly reduced in intensity indicating that the O-antigen is either truncated (“rough mutant”) or the O-antigen repeat units were present at much lower concentration (Figure 2).

## Discussion

We have engineered a novel R-type pyocin, AvR2-O104.1, which is highly specific and sensitive for *E. coli* O104. We conclude that the engineered pyocin utilizes the O104-antigen as a receptor for the following reasons. (i) Related phage P22-like tail spikes typically target O-antigens or K antigens. (ii) All of the strains that produce the O104 antigen were AvR2-O104.1-sensitive, regardless of other antigenic determinants expressed. (iii) German outbreak strain RM15013 expressing a highly truncated O104 LPS (i.e. “rough mutant”) was resistant to AvR2-O104.1. (iv) AvR2-O104.1-resistant mutants selected from other O104 strains also had truncated O104-antigens. (v) A K9 strain of *E. coli* expressing the same repeating structural polysaccharide unit as O104 also was sensitive to AvR2-O104.1. (vi) *E. coli* strains expressing other O-antigen serotypes were not sensitive to AvR2-O104.1.

In the presence of high concentrations of R-type pyocins, resistant mutants can be selected. This was noted for the O157-specific pyocin AvR2-V10.3 [15] and is also the case for AvR2-104.1. For both pyocins, the dominant resistant phenotype is loss of high molecular weight O-antigen. Since O-antigens typically contribute to virulence [18], [19] we expect that these resistant mutants are compromised in virulence and are expected to exhibit concomitant reduction of pathogenic threat.

The tailspike gene used to create AvR2-O104.1 is likely part of a prophage remnant in the genome of the German outbreak strain, since there is a deletion in nearby prophage genes, D-ROD4 [10]; whereas, in the close relative enteroaggregative O104:H4 strain 55989 [20], this locus appears to encode a complete prophage. However, it has not been determined whether this latter locus is capable of producing infectious particles. Phage P22-like tailspike proteins are responsible for host specificity and typically bind to O-antigens. These tailspike proteins consist of a highly conserved N-terminal head-binding domain of about 110 amino acids and a C-terminal O-antigen binding domain that is divergent [21]–[22]. Consistent with this structure, the first 113 amino acids of the *E. coli* O104 tail spike share 79% sequence identity with that of the phage P22 tailspike, whereas the C-terminal 546 amino acids share little sequence similarity with anything in the GenBank database. However, based on the fact that the C-terminal portion of tail spikes of similar phages are responsible for receptor binding, we reasoned that the comparable region of this protein has a similar function. The killing spectrum of AvR2-O104.1 confirms that this C-terminal domain is in fact responsible for O-antigen binding.

P22-like prophages typically encode an acetylase gene that modifies the O-antigen [23]–[25]; acetylation of the O-antigen of an O104 strain has been reported previously [26]. Indeed, a putative O-antigen acetylase gene, EGR74046 (Genbank accession no. AFOB02000092), located just downstream of the tailspike gene in both the German outbreak strain and enteroaggregative O104:H4 strain 55989 could be responsible for this O-antigen acetylation in both strains. The fact that the acetylase gene is retained in the prophage remnant of the German outbreak strain in spite of its losing much of the prophage locus is intriguing and worthy of determining functional activity, especially considering the hyper-virulence of the German O104:H4 outbreak strains.

Natural R-type pyocins have been shown in animal models to be efficacious in treating systemic infections [27]–[29]. More recently, the engineered R-type pyocin AvR2-V10.3 specific for *E. coli* O157 strains has been demonstrated to be efficacious at treating *E. coli* O157:H7 infections in an infant rabbit model [16]. We anticipate that a panel of four to six engineered pyocins targeting specific STECs associated with the most severe disease (e.g. HUS), and now including AvR2-O104, will be a valuable tool for treating these serious infections.

A recent commentary in *Nature* emphasized the failure of genomics to advance the development of new treatments for bacterial diseases [30]. In that context, it is worth noting that the published draft unassembled genome sequence data from the *E. coli* O104:H4 strain responsible for the 2011 German outbreak, provided rapidly by next generation DNA sequencing technology, facilitated identification of a strain-specific binding motif in the form of a tailspike of a presumably defective prophage. This information led directly to the development of a targeted bactericidal agent to kill O104-positive *E. coli*. Thus, this R-type pyocin platform strategy provides at least one example of the potential value of a draft sequence for the design and production of specific bactericidal agents against emerging bacterial pathogens of humans. A recent BLAST search for P22-like tailspikes shows that several dozen sequenced *E. coli* genomes encode P22-like tailspikes, which could immediately be used to generate specific pyocins. Furthermore, other phage tail fiber genes have been used to retarget pyocins [14] and it is likely that nearly every *E. coli* genome encodes some prophage or prophage remnant receptor binding protein. Of note is the speed in which a specific R-type pyocin can be developed. Upon determination of the genome sequence of a new pathogen, it would be a matter of weeks to generate a pyocin by this method; this time could be cut to less than one week if DNA from the strain is available for PCR amplification.

## Materials and Methods

### Strains

Table 1 shows strains tested for sensitivity to AvR2-O104.1. Clinical isolates of O104:H4 were kindly provided by: J. Rudrick, Michigan Dept. of Public Health (RM15012, RM15013, RM15014); F. Valdes, Ontario Agency for Health Protection and Promotion (RM15031); L. Connolly, Massachusetts Dept. of Public Health (RM14735); M. McDaniels, N. Strockbine, E. Sowers, Centers for Disease Control (RM15038, RM15039); J. Nataro and F. Ruiz, U. of Maryland (RM15037). All other strains were obtained from the culture collection maintained by R. Mandrell at the USDA, ARS, WRRC, Produce Safety and Microbiology Research Unit, Albany, CA. Patient sources of strains are anonymous. IRB approval is not required for testing of clinical bacterial isolates.

### Construction of AvR2-104.1 and AvR2-104.2

The entire tailspike gene from the remnant prophage of *E. coli* O104:H4 was synthesized by GenScript (Piscataway, NJ). From this template, PCR primers were designed to create genes encoding fusions between the C-terminus of the tailspike and the N-terminal amino acid residues 1–164 of the R2 tail fiber. Plasmid DG100, which contains the R2-P2 tail fiber fusion under control of P_TAC_ [14], was digested with EcoR1 and HindIII to excise the C-terminal portion of the P2 tail fiber. Two separate PCR fragments encoding amino acids 114–660 (primers 5′-tttcctaagcttcggcaagaattacaggggag-3′ and 5′- aaacgacggccagtgaattc -3′) or 118–660 (primers 5′-tttcctaagcttcaggggagcgcaggagtcat -3′ and 5′- aaacgacggccagtgaattc-3′) of the *E. coli* O104 tail spike were amplified with HindIII and EcoR1 ends and inserted into the digested DG100 to create DG645 and DG646 respectively. These plasmids were transformed into PAO1 Δ*prf15* [14] to create BDG63 and BDG64 respectively.

### Expression of AvR2-104.1 and AvR2-104.2

BDG63 and BDG64 were grown at 37°C in trypticase soy broth supplemented with 100 µg/ml gentamicin to an OD600 of 0.3 cm^−1^, after which IPTG and mitomycin C were added to 250 µM and 3 µg per ml, respectively. After 5 hours the culture was visibly lysed (OD600 of <0.2 cm^−1^). Large cellular debris was removed by centrifugation at 6000×g for 20 minutes. Finer cellular debris was removed by centrifugation at 16,000×g for 1 hour. This was followed by a high speed spin at 42,000×g for 1 hour to pellet the pyocin particles. Pyocin in the pellet was typically resuspended in 10 mM Tris-Cl pH 7.5, 50 mM NaCl at a volumes of 1/200 to 1/50 of the lysate volume.

### Activity assays

Spot killing assays were performed as described previously [14]. Briefly, lawns of target bacteria were prepared by adding 5 ml of molten tempered LB agar (0.5%) to 200 µl of an overnight culture. This inoculated overlay agar was poured onto a dry 1.5% LB agar plate and allowed to set. Aliquots (5 µl) of serial 5-fold dilutions of pyocin were spotted directly on the top agar and allowed to dry. After overnight incubation, killing of target bacteria was indicated by circular zones of clearing in the lawn of bacterial growth. Titration survival assays were performed also as described previously [14]. Briefly, dilutions of pyocin are added to a fixed number of bacteria (1×10^9^ cfu), incubated for 40 min at 37°C, then plated to count bacterial survivors. The concentration of pyocin is measured as individual bactericidal killing units (KU) which is related to the fraction of survivors in a Poisson distribution *m* = −ln*S* where *m* is the average number of killing events per cell and *S* is the fraction of bacterial survivors.

### SDS-PAGE analysis of LPS

LPS was prepared and analyzed by SDS-PAGE by the following procedure. Bacteria grown on Luria-Bertani agar overnight were harvested with a 10 µl loop and suspended in 200 µl sterile water in a microfuge tube. A 200 µl sample of freshly prepared 2× Tricine gel sample buffer (cat. no. 161-0739; Bio-Rad, Hercules, CA) containing a final concentration of 286 mM 2-mercaptoethanol (Sigma-Aldrich, cat no. M-138, 14.3 M stock) (TB-ME) was added to each sample, the sample mixtures were vortexed for 1 minute, then incubated in boiling water for 5 minutes to lyse bacteria. After cooling to room temperature, 10 µl of a 20 mg ml^−1^solution of Proteinase K (PK) (Roche Inc.; recombinant Proteinase K) was added and the samples were incubated in a 60°C water bath for 2 hr to digest proteins in the lysate. The PK-treated lysates can be used immediately or stored at 4°C until needed. A 25 µl sample of this sample was transferred to a new microfuge tube and 75 µl of fresh TB-ME was added and the samples were heated at 100°C for 2 min. Five µl samples were added to wells of a 10–20% Tricine gradient gel (Invitrogen, Inc., Cat no. EC6625BOX), and the gels were electrophoresed in Tricine SDS running buffer (Invitrogen Novex, Cat no. LC1675) in an electrophoresis system (Invitrogen/Life Tech., Novex Xcell Surelock) for 70 min at 135 constant voltage (BioRad Powerpak 200; Hercules, CA). The gels were treated with sodium metaperiodate and LPS was silver stained as described previously [31].
